# Childhood Behavioural Problems and Adverse Outcomes in Early Adulthood: a Comparison of Brazilian and British Birth Cohorts

**DOI:** 10.1007/s40865-019-00126-3

**Published:** 2019-10-27

**Authors:** Gemma Hammerton, Joseph Murray, Barbara Maughan, Fernando C. Barros, Helen Gonçalves, Ana Maria B. Menezes, Fernando C. Wehrmeister, Matthew Hickman, Jon Heron

**Affiliations:** 1 Population Health Sciences, University of Bristol, Bristol, UK; 2 Postgraduate Program in Epidemiology, Universidade Federal de Pelotas, Pelotas, Brazil; 3 MRC Social, Developmental and Genetic Psychiatry Centre, Institute of Psychiatry, Psychology & Neuroscience, King’s College London, London, UK

**Keywords:** ALSPAC, Pelotas, Behavioural problems, Brazil, Measurement invariance

## Abstract

**Purpose:**

Examine associations between childhood behavioural problems with criminal behaviour, emotional disorders, substance use and unemployment in early adulthood in two birth cohorts from a middle- and high-income country.

**Methods:**

Data were utilised from large, prospective birth cohorts in Brazil (1993 Pelotas Birth Cohort; *N* = 3939) and the UK (Avon Longitudinal Study of Parents and Children; ALSPAC; *N* = 5079). Behavioural problems were reported on by parents at age 11 years (including disobeys, temper, lies, fights, steals). Outcomes (assessed with youth between ages 22 and 24 years) included criminal behaviour, emotional disorders, substance use and NEET (not in education, employment or training).

**Results:**

In both cohorts, children with ‘conduct problems’ (those with increased probability of all five behaviours at age 11), were at higher risk of criminal behaviour, emotional disorders and NEET in adulthood compared to those with ‘low problems’. Associations for ‘conduct problems’ were stronger in Pelotas compared to ALSPAC for hazardous alcohol use [Pelotas: risk ratio = 1.39, 95% CI = 1.14–1.70; ALSPAC: risk ratio = 0.76, 95% CI = 0.57–1.02] and illegal drug use [Pelotas: risk ratio = 1.32, 95% CI = 1.16–1.50; ALSPAC: risk ratio = 1.05, 95% CI = 0.91–1.20], whereas associations for criminal behaviour [Pelotas: risk ratio = 1.92, 95% CI = 1.29–2.86; ALSPAC: risk ratio = 2.75, 95% CI = 2.04–3.73] and NEET [Pelotas: risk ratio = 1.38, 95% CI = 1.13–1.70; ALSPAC: risk ratio = 3.04, 95% CI = 1.99–4.65] were stronger in ALSPAC.

**Conclusions:**

Childhood conduct problems were associated with criminal behaviour, emotional disorders and unemployment in adulthood in both Brazil and the UK. Additional associations were found for substance use in Brazil.

**Electronic supplementary material:**

The online version of this article (10.1007/s40865-019-00126-3) contains supplementary material, which is available to authorized users.

## Introduction

Behavioural problems are common in childhood and include both behaviours related to conduct disorder (CD; norm-breaking behaviours and violations of the rights of others) and behaviours related to oppositional defiant disorder (ODD; noncompliant, angry and defiant behaviours). There is increasing evidence from prospective, longitudinal studies in high-income countries (HICs) that these behavioural problems, particularly CD, are associated with a wide range of adverse outcomes in adulthood e.g. [1–3]. Both these early behaviour problems and their adverse outcomes have considerable financial and emotional impact on affected young people and their families, as well as on education, mental health and juvenile justice systems.

Although the adverse outcomes associated with childhood behavioural problems are well-established in HICs, much less is known about the long-term consequences of behavioural problems in low- and middle-income countries (LMICs). This is especially important to address in LMICs with high levels of behavioural problems and crime, such as Brazil, where processes underlying the associations may differ from HICs [4, 5]. Brazil has one of the highest rates of inequality in the world with the 19th highest GINI out of 158 countries, while the UK ranked 117th in 2014 [6]. In 2018, the youth (age 15 to 24 years) unemployment rate was 28.5% in Brazil compared to 12.1% in the UK [6]. Brazil also has the highest years of life lost to violence out of any WHO member state [7]. However, national surveys show higher rates of drug use, particularly cannabis, in the UK compared to Brazil [5].

It is possible that childhood behavioural problems would have more impact in Brazil, as the average child will have fewer protective support systems, and more stressors interacting with behavioural problems leading to adverse outcomes. Therefore, it cannot be assumed that associations found in HICs will be the same across contexts. Previous studies comparing Brazil to the United Kingdom (UK) have found higher levels of childhood behavioural problems in Brazil [4, 8]. However, behavioural problems were predicted by similar early-life biological and sociodemographic risk factors across countries [9] and carried similar risk for crime at age 18 years after adjusting for these early-life risk factors [8]. No study that we are aware of has examined whether childhood behavioural problems carry similar risk (across Brazil and the UK) for a wider range of adverse outcomes in adulthood.

It is currently unclear whether the higher levels of childhood behavioural problems found in Brazil compared to the UK reflect genuine differences or simply reflect Brazilian mothers over reporting their child’s behavioural problems [4, 9]. Even when the same questionnaire measures are used across cohorts, biases can arise due to translation and differences in responding across contexts [10, 11]. These biases can be addressed at the analysis stage using psychometric modelling techniques to test for measurement invariance (i.e. the extent to which a set of items assesses an underlying construct in a similar way across cohorts).

Previous research suggests different possible outcomes of childhood behaviour problems depending on their sub-type, with those related to CD (such as stealing and fighting) predicting an increased risk for criminal behaviour, mental health and substance use problems and unemployment [1–3, 12], and behaviours related to ODD (such as temper outbursts) mainly predicting an increased risk for later emotional disorders in both HICs [12–14] and Brazil [15]. In the current study, rather than examining actual clinical behavioural disorders, we examine levels of behaviours related to each of CD and ODD assessed using a questionnaire measure. The main aim of the study is to examine the adverse consequences of childhood ‘conduct problems’ (such as fighting, lying and stealing) and ‘oppositional problems’ (such as losing temper and disobedience) across Brazil and the UK.

In summary, we aim to advance knowledge on the important negative outcomes of childhood behaviour problems by (i) testing for comparability of childhood behavioural problems across the UK and Brazil using two well-matched population-based birth cohorts to derive equivalent latent classes (or groups) representing ‘conduct problems’, ‘oppositional problems’, and ‘low problems’; (ii) establishing associations between these behavioural problems and criminal behaviour, emotional disorders, substance use and unemployment in early adulthood in a middle-income setting (Brazil) and (iii) investigating whether the strength of associations are similar to those found in a high-income setting (UK). We hypothesise that childhood conduct problems will be strongly associated with all adverse outcomes, but that childhood oppositional problems will primarily show associations with emotional disorders. We also hypothesise that associations will be generally similar across cohorts, but with a weaker magnitude of effect when the outcome reflects a more normative behaviour in one context (e.g. substance use in early adulthood is more normative in the UK compared to Brazil).

## Method

### Samples

#### 1993 Pelotas Birth Cohort Study, Brazil

The 1993 Pelotas Birth Cohort Study is an ongoing population-based study designed to investigate the effects of a wide range of influences on health and development. Pelotas is a city located in the extreme south of Brazil, with an estimated population of 345,179 inhabitants, 93% of whom live in the urban area. All births occurring in the five maternity clinics in the town were monitored in 1993 (99% of births in Pelotas occurred in hospital). For the 5265 children born alive, only 16 mothers could not be interviewed or refused to participate in the study. The 5249 newborns, whose mothers lived in the urban area, were included in the cohort (81 were either twins or triplets). The detailed methodology of this study can be found elsewhere [16]. During the perinatal study, mothers were interviewed to collect demographic, health and socioeconomic information about the family. Follow-up home visits were conducted in 2004–2005 (age 11; *N* = 4452 mothers; 85% of original cohort) and in clinic sessions in 2015–2016 (age 22; *N* = 3810 young people; 73% of original cohort) [17, 18]. Study data at age 22 years was collected and managed using REDCap electronic data capture tools [19]. The perinatal study and each follow-up were approved by the Research Ethics Committee of the Federal University of Pelotas School of Medicine. After being informed of the details of the study, participants signed a term of informed consent.

### Avon Longitudinal Study of Parents and Children (ALSPAC), UK

ALSPAC is an ongoing birth cohort which was set up to examine genetic and environmental determinants of health and development [20]. ALSPAC recruited pregnant women resident in Avon, UK with expected dates of delivery 1st April 1991 to 31st December 1992. Of the 14,541 initial pregnancies, there was a total of 14,676 foetuses, resulting in 14,062 live births and 13,988 children who were alive at 1 year of age (of which 179 were twins). Parents and children have been followed up regularly since recruitment via questionnaire and clinic assessments. In the current study, data were used from follow-ups with mothers in pregnancy and perinatally (*N* = 13,545 to 9638; 97 to 69% of original cohort) and when their child was aged 11 years (*N* = 7912; 57% of original cohort), and follow-ups with young people at age 22 years (*N* = 4026; 29% of original cohort) and 24 years (*N* = 4026; 29% of original cohort). Study data from 2014 onwards were collected and managed using REDCap electronic data capture tools hosted at University of Bristol [19]. Further details on the sample characteristics and methodology have been described previously [20, 21], and detailed information about ALSPAC can be found on the study website (http://www.bristol.ac.uk/alspac). For information on all available ALSPAC data see the fully searchable data dictionary (http://www.bristol.ac.uk/alspac/researchers/our-data/). Written, informed consent was obtained from all mothers who entered the ALSPAC study, and ethical approval for the study was obtained from the ALSPAC Ethics and Law committee and the Local Research Ethics Committees.

### Measures

#### Exposure: Behavioural Problems at Age 11 Years

Behavioural problems were assessed at age 11 years using the parent-rated five-item conduct problems subscale (range 0–10) of the Strengths and Difficulties Questionnaire (SDQ) [22]. The SDQ is a screening questionnaire that assesses child mental health symptoms in the previous 6 months, and it has been validated in both the UK [23] and Brazil [24, 25] using independently diagnosed psychiatric disorders. Items include indicators of both CD and ODD, including, ‘often has temper tantrums or hot tempers’, ‘generally obedient, usually does what adults request’, ‘often fights with other children or bullies them’, ‘often lies or cheats’ and ‘steals from home, school or elsewhere’.

#### Outcomes at Ages 22 to 24 Years

##### Criminal behaviour

In both studies, criminal behaviour was assessed using a self-report questionnaire referring to crimes committed in the previous year, originally developed in the Edinburgh Study of Youth Transitions and Crime [26]. External validity for this self-report questionnaire has been examined previously in adolescents using cross-checks with official records and teachers’ questionnaires in the UK [27] and in relation to official records in Brazil. In Pelotas, official police, court and juvenile justice institution data were collected, and a previous study found a strong association (risk ratio = 5.2) between self-reported violence and having an official record of violent crime committed at age 18 [9]. A binary variable was created representing any crime (including stole from shops/stores, damaged property, stole from vehicle, stole vehicle, sold drug, burgled, sold stolen goods, arson, stole from person (with or without use of force), assault or carried a weapon for fights or self-defence) committed in the previous year. In Pelotas, questions were translated into Brazilian Portuguese, then pilot tested among adolescent offenders and adolescents in the community, adjusted by bilingual researchers, further pilot tested and then included in confidential questionnaires at age 22 years. In ALSPAC, criminal behaviour was assessed at age 24 years during a computer-based session at a focus clinic.

##### Emotional disorders

In Pelotas, DSM-5 major depressive disorder (MDD) and generalized anxiety disorder (GAD) were assessed at age 22 years using the Mini-International Neuropsychiatric Interview (MINI [28]). The MINI is a diagnostic interview that has been validated in Brazil using independently diagnosed psychiatric disorders [29]. It was administered by trained psychologists who were blind to exposure status. In ALSPAC, DSM-5 MDD and ICD-10 GAD were assessed at the focus clinic at age 24 years using the computerised version of the Clinical Interview Schedule Revised (CIS-R [30]).

##### Substance use

In both studies, hazardous alcohol use was assessed using the self-report 10-item Alcohol Use Disorders Identification Test (AUDIT [31]) which is a brief screening tool to identify individuals with alcohol-related problems. The AUDIT scale has been studied extensively and has high validity and reliability in the detection of risky drinking and alcohol dependence [32] and has been validated using diagnostic interview, physical examinations and laboratory testing [33]. The AUDIT was dichotomised at a cut-point of eight and treated as a binary variable. This cut-point represents hazardous levels of drinking and has been validated both in the UK [34] and Brazil [35] using psychiatric diagnoses of alcohol use disorders. Alcohol use was assessed using a self-report questionnaire at age 22 years.

Illicit drug use was assessed using the same self-report questionnaire at age 22 years which included questions about the lifetime use of cannabis, cocaine, crack, amphetamine-type stimulants, nitrous oxide or other inhalants, hallucinogens, opioids and other injected illegal drugs. A binary variable was created in each study representing the lifetime use of any illegal drug.

##### Not in education, employment or training (NEET)

In both studies, NEET was assessed using the same self-report questionnaire at age 22 years which also included two questions on whether participants were currently enrolled in any education/training programme or employed. A binary variable was created which classified the young person as NEET if the answer to both questions was negative, in line with the definition used by the Office for National Statistics [36].

#### Potential Confounders

Data on biological and sociodemographic factors were collected during perinatal assessments with mothers in Pelotas and during pregnancy and perinatal assessments with mothers in ALSPAC. Biological factors included unplanned pregnancy (yes/no), mother ever smoked in pregnancy (yes/no), mother used alcohol in pregnancy (yes/no), maternal urinary infection during pregnancy (yes/no), intrauterine growth restriction (yes/no; referring to < 10th percentile/≥ 10th percentile for gestational age and gender, according to the reference curve developed by Kramer and colleagues [37] and premature birth < 37 weeks (yes/no). The cumulative number of biological risk factors was summed, up to six, for each child as has been done previously [9].

Sociodemographic factors included maternal age (< 20 years/≥ 20 years), low maternal education (yes/no; referring in Pelotas to 0**–**8 vs. ≥ 9 years of schooling and referring in ALSPAC to qualified up to certificate of secondary qualification level, vs. qualified to at least vocational level, O-level or A-level), marital status (single mother/with partner), three or more siblings (yes/no) and family income (lowest quintile/second-fifth quintiles). Again, the cumulative number of sociodemographic risk factors was summed, up to five, for each child as has been done previously [9].

Maternal depression was assessed with mothers in Pelotas when their children were aged 11 years using the Self Report Questionnaire (SRQ), which has been validated in Brazil [38]. A cut-off of eight was used to represent probable minor psychiatric disorder. In ALSPAC, maternal depression was assessed with mothers when their children were aged 11 years using the 10-item Edinburgh Postnatal Depression Scale (EPDS [39]); with a cut-off of 13 points representing probable depression).

Parental separation was assessed in Pelotas at age 11 years using a child-report question about their parent’s divorce and a mother-report question about whether the child’s biological father lives in the house. Child-report of parental divorce or mother-report of biological father not living in the house were both classed as parental separation. In ALSPAC, parental separation was assessed using multiple mother-report questions between child age 8 months and 11 years. A binary variable was created representing any reports of divorce or separation from mother’s partner between the child’s birth and age 11 years.

Finally, fear of the neighbourhood was assessed in Pelotas using a mother-report question asking whether she was afraid of living in the neighbourhood (yes/no) when the child was aged 11 years. In ALSPAC, fear of the neighbourhood was assessed using a mother-report questionnaire at child age 10 years asking about whether the mother was worried about vandalism, muggings and robberies in the neighbourhood. Any reports of vandalism, muggings or robberies as a minor or serious problem were combined to create a binary variable representing fear of the neighbourhood.

## Statistical Analysis

### Latent Class Analysis (LCA) for Behavioural Problems at Age 11 Years

LCA was performed with the five binary items measuring behavioural problems as latent class indicators. LCA was employed both to minimise measurement error in the assessment of childhood behavioural problems, but also to enable us to test for measurement invariance across studies. To test for measurement invariance of behavioural problems in the context of LCA, a stepwise multiple indicator multiple cause model was used [40]. The steps that were carried out (from the initial derivation of the latent classes to the validation of the final classes in each cohort after allowing partial measurement invariance) are detailed in Online Resource 1.

### Associations Between Latent Classes of Behavioural Problems and Adverse Outcomes at Ages 22 to 24 Years

**Fig. 1:**
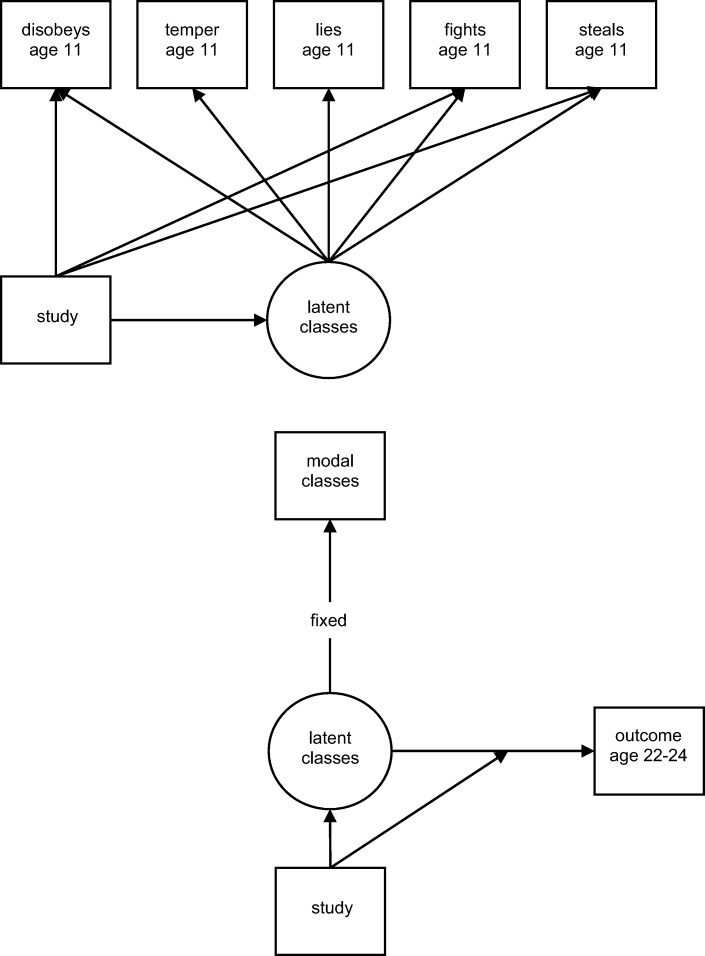
Path diagram for the measurement and analysis model. Top: Path diagram for the measurement model (derivation of latent classes) showing uniform differential item functioning (DIF) effects of study on ‘disobeys’, ‘fights’, and ‘steals’. Bottom: Path diagram for the analysis model showing bias-adjusted three -step methods to relate the latent classes to a binary distal outcome; confounders not shown on figure

Associations between the latent classes and the binary distal outcomes were estimated using a bias-adjusted three-step approach. The modal class assignment from the final three-class model with partial measurement invariance across study was used as a nominal indicator of the latent class variable with the study-specific, class-specific multinomial intercepts fixed at values corresponding to the estimated error rates [41]. This method accounts for the uncertainty in latent class assignment but prevents the shift in class distributions that is often seen using a one-step approach. In all analyses, the ‘low class’ is treated as the reference class and associations are reported as risk ratios (with 95% confidence intervals). A path diagram is shown in Fig. 1. All models were analysed in Mplus v8 using maximum likelihood estimation [42].

### Missing Data

For the derivation of latent classes, missing data were handled using full information maximum likelihood (FIML) estimation [43]. In FIML estimation, all available data are used and the likelihood (joint probability of observed data given the parameters) for each ‘missing data pattern’ is calculated, summing (or integrating) over the variables with missing data. Any respondent with at least one item assessing behavioural problems complete at age 11 years (Pelotas: *N* = 4423; ALSPAC: *N* = 7076) was included in the LCA under the missing at random assumption (i.e. there are no systematic differences between observed and missing values for any dependent variable when conditioning on the remaining variables in the model)*.* Those with missing outcome data were also included in the analysis sample using FIML estimation; however, the analysis sample was restricted to those with complete data available for the confounding factors (Pelotas: *N* = 3939; ALSPAC: *N* = 5079). A flowchart of retention in both studies is shown in Online Resource 2.

## Results

### Latent class Analysis for Behavioural Problems at Age 11 Years

**Fig. 2:**
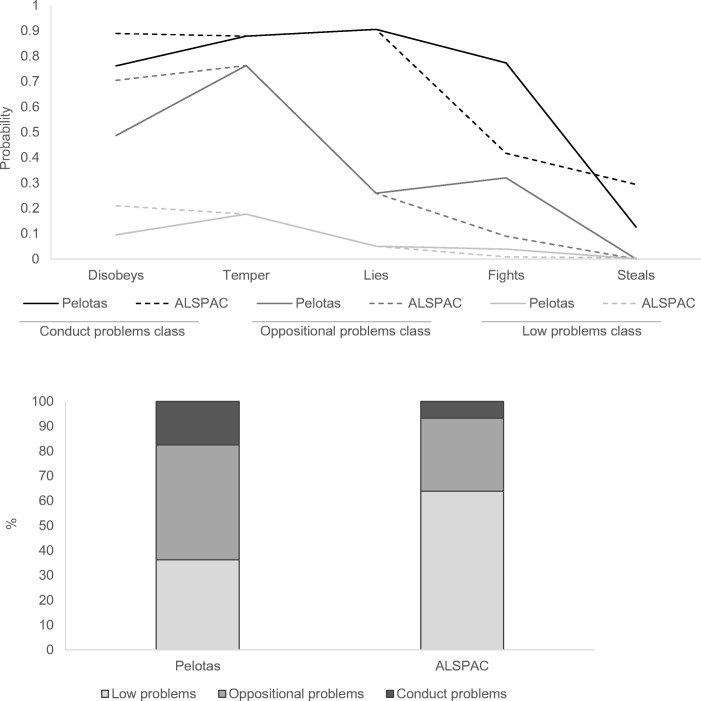
Measurement and structural model results based on the final three-class multiple indicator multiple causes (MIMIC) latent class model with partial measurement invariance across study. Top: Class profile plots showing uniform differential item functioning (DIF) effects of study on ‘disobeys’, ‘fights’, and ‘steals’. Bottom: Model estimated latent class variable distribution by study

One- to five-class models were compared for the latent classes of behavioural problems. The three-class model provided the best fit to the data, and partial measurement invariance across study was found (Online Resource 1). The measurement and structural model results based on the final three-class model with partial measurement invariance are shown in Fig. 2 and validation of the latent classes is provided in Online Resource 1.

In Pelotas, 18% of the sample were classified as having a high probability of all items (referred to throughout as the ‘conduct problems’ class), and 46% were classified as having a high probability of items related to oppositional problems (referred to throughout as the ‘oppositional problems’ class). In ALSPAC, 7% of the sample were classified as having a high probability of all items (‘conduct problems’ class), and 29% were classified as having a high probability of endorsing items related to oppositional problems. Associations between potential confounders and latent classes of behavioural problems are shown in Online Resource 3.

### Associations Between Latent Classes of Behavioural Problems and Adverse Outcomes at Ages 22 to 24 Years

The prevalence of criminal behaviour in early adulthood was higher in ALSPAC compared with Pelotas (13% vs. 10%; *p* < 0.001). Specifically, the prevalence of non-violent crime (including stole from shops/stores, damaged property, stole from vehicle, stole vehicle, sold drug, burgled, sold stolen goods, arson and stole from person without use of force) was higher in ALSPAC (10% vs. 3%), whereas, the prevalence of violent crime (including stole from person with use of force, assault and carried a weapon for fights or self-defence) was higher in Pelotas (8% vs. 5%). The prevalence of MDD (9% vs. 5%; *p* < 0.001), hazardous alcohol use (42% vs. 21%; *p* < 0.001) and illicit drug use (59% vs. 39%; *p* < 0.001) was also higher in ALSPAC compared with Pelotas. The prevalence of GAD was higher in Pelotas compared with ALSPAC (16% vs. 9%; *p* < 0.001), as was the prevalence of NEET (22% vs. 9%; *p* < 0.001).

**Fig. 3:**
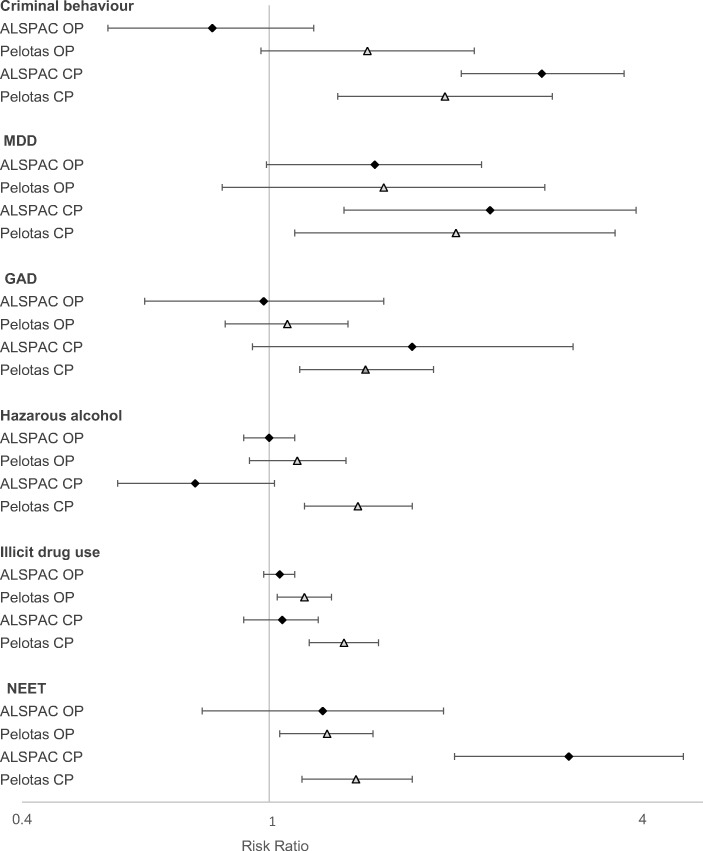
Associations between the latent classes of behavioural problems at age 11 years and adverse outcomes in early adulthood after adjusting for biological and sociodemographic factors, child sex, maternal depression, parental separation and fear of the neighbourhood; showing risk ratio (95% confidence interval) with ‘low problems’ as the reference group; diamond represents ALSPAC; triangle represents Pelotas; OP, oppostional problems; CP, conduct problems

In both cohorts, after adjusting for potential confounders, those with childhood ‘conduct problems’ were at higher risk of criminal behaviour (Pelotas: risk ratio = 1.92, 95% CI = 1.29–2.86; ALSPAC: risk ratio = 2.75, 95% CI = 2.04–3.73), MDD (Pelotas: risk ratio = 2.00, 95% CI = 1.10–3.61; ALSPAC: risk ratio = 2.27, 95% CI = 1.32–3.90) and NEET (Pelotas: risk ratio = 1.38, 95% CI = 1.13–1.70; ALSPAC: risk ratio = 3.04, 95% CI = 1.99–4.65) in adulthood compared to those with ‘no problems’. There was also strong evidence in Pelotas that ‘conduct problems’ were associated with increased risk for GAD (Pelotas: risk ratio = 1.43, 95% CI = 1.12–1.84; ALSPAC: risk ratio = 1.70, 95% CI = 0.94–3.09), hazardous alcohol use (Pelotas: risk ratio = 1.39, 95% CI = 1.14–1.70; ALSPAC: risk ratio = 0.76, 95% CI = 0.57–1.02) and illicit drug use (Pelotas: risk ratio = 1.32, 95% CI = 1.16–1.50; ALSPAC: risk ratio = 1.05, 95% CI = 0.91–1.20) (Fig.3).

There was little evidence of an association between ‘oppositional problems’ (compared to ‘low problems’) and the adverse outcomes, except for weak associations for illicit drug use (Pelotas: risk ratio = 1.14, 95% CI = 1.03–1.26; ALSPAC: risk ratio = 1.04, 95% CI = 0.98–1.10) and NEET (Pelotas: risk ratio = 1.24, 95% CI = 1.04–1.47; ALSPAC: risk ratio = 1.22, 95% CI = 0.78–1.91) in Pelotas.

There was evidence for an interaction between study and the latent classes of behavioural problems (joint test for both ‘conduct problems’ and ‘oppositional problems’ versus ‘low problems’) in predicting criminal behaviour (*p* = 0.003), NEET (*p =* 0.003), hazardous alcohol use (*p* = 0.004) and illicit drug use (*p* = 0.047). There was no evidence for an interaction between study and the latent classes of behavioural problems in predicting MDD (*p* = 0.925) or GAD (*p* = 0.807).

Unadjusted associations were similar to adjusted and both are shown in Table 1. Results after additionally adjusting for comorbidity at age 11 years (emotional and hyperactivity problems) were slightly weaker (Online Resource 4).

**Table 1:** Unadjusted and adjusted associations between the latent classes of behavioural problems at age 11 years and adverse outcomes at ages 22 to 24 years; showing risk ratio (95% confidence interval) with ‘low problems’ as the reference group

	Pelotas (*N* = 3939)					ALSPAC (*N* = 5079)					Interaction^1^
Model 1 (unadjusted)	*n*	%	Oppositional problems	Conduct problems	*p* value	*n*	%	Oppositional problems	Conduct problems	*p* value	*p* value
Criminal behavioural	382	10	1.67 (1.03–2.71)	2.64 (1.72–4.06)	< 0.001	676	13	0.50 (0.23–1.11)	3.84 (2.76–5.33)	< 0.001	0.010
MDD	181	5	1.71 (0.88–3.34)	2.56 (1.41–4.66)	0.009	437	9	1.65 (0.98–2.80)	2.67 (1.38–5.19)	0.001	0.457
GAD	642	16	1.11 (0.82–1.52)	1.68 (1.27–2.21)	< 0.001	452	9	0.99 (0.55–1.76)	1.94 (0.97–3.88)	0.158	0.883
Hazardous alcohol use	835	21	1.10 (0.85–1.43)	1.52 (1.20–1.92)	0.002	2153	42	1.06 (0.88–1.27)	0.64 (0.39–1.05)	0.213	0.002
Illicit drug use	1528	39	1.20 (1.00–1.43)	1.47 (1.24–1.73)	< 0.001	2992	59	1.09 (0.96–1.25)	1.05 (0.81–1.35)	0.333	0.074
NEET	851	22	1.37 (1.05–1.78)	1.76 (1.38–2.25)	< 0.001	437	9	1.12 (0.59–2.15)	4.03 (2.53–6.43)	< 0.001	< 0.001
Model 2 (adjusted^2^)	*n*	%	Oppositional problems	Conduct problems	*p* value	*n*	%	Oppositional problems	Conduct problems	*p* value	*p* value
Criminal behavioural	382	10	1.44 (0.97–2.14)	1.92 (1.29–2.86)	0.006	676	13	0.81 (0.55–1.18)	2.75 (2.04–3.73)	< 0.001	0.003
MDD	181	5	1.53 (0.84–2.78)	2.00 (1.10–3.61)	0.073	437	9	1.48 (0.99–2.20)	2.27 (1.32–3.90)	0.004	0.925
GAD	642	16	1.07 (0.85–1.34)	1.43 (1.12–1.84)	0.011	452	9	0.98 (0.63–1.53)	1.70 (0.94–3.09)	0.212	0.807
Hazardous alcohol use	835	21	1.11 (0.93–1.33)	1.39 (1.14–1.70)	0.005	2153	42	1.00 (0.91–1.10)	0.76 (0.57–1.02)	0.172	0.004
Illicit drug use	1528	39	1.14 (1.03–1.26)	1.32 (1.16–1.50)	< 0.001	2992	59	1.04 (0.98–1.10)	1.05 (0.91–1.20)	0.446	0.047
NEET	851	22	1.24 (1.04–1.47)	1.38 (1.13–1.70)	0.006	437	9	1.22 (0.78–1.91)	3.04 (1.99–4.65)	< 0.001	0.003

^1^Interaction represents whether study (Pelotas vs ALSPAC) modifies the association between behavioural problems and each adverse outcome; ^2^Adjusted for biological and sociodemographic factors, child sex, maternal depression, parental separation and fear of the neighbourhood; *MDD*, major depressive disorder; G*AD*, generalized anxiety disorder; *NEET*, not in education, employment or training; *n* (%) for each adverse outcome based on estimated model using FIML estimation

## Discussion

### Summary of Findings

Even after accounting for partial measurement invariance of childhood behavioural problems across study, the prevalence of parent-reported conduct problems (18% vs. 7%) and oppositional problems (46% vs. 29%) was still higher in Brazil compared to the UK. In Brazil, we found that children with conduct problems (those whose mothers had a high probability of endorsing the five behaviours that were assessed at age 11) were at increased risk for criminal behaviour, emotional disorders, substance use and NEET in early adulthood compared to those with low problems. Associations were generally similar across contexts; however, associations for conduct problems with criminal behaviour and NEET were stronger in the UK compared to Brazil, while associations for hazardous alcohol use and illegal drug use were only found in Brazil. Few associations were found for childhood oppositional problems (compared to low problems) except for weak associations for illicit drug use and NEET in Brazil.

### Strengths and Limitations

We examined associations between childhood behavioural problems and adverse outcomes spanning multiple domains across a period of more than 10 years using two large, prospective, population-based cohorts in a middle- and high-income setting that are well-matched in terms of study design, year at birth, ages at follow-up and instruments used to assess the exposure and outcomes. All biological and sociodemographic variables in the perinatal period and maternal depression have been carefully compared between Pelotas and ALSPAC in previous studies [8, 9, 44]. Associations between childhood behavioural problems and later criminal behaviour have also been compared previously across cohorts [8, 9]. We also tested for measurement invariance of behavioural problems to ensure that the latent classes derived were comparable across study. This is important because ignoring potential variation in responding across cohorts results in ambiguity regarding whether any cross-context differences in the construct of interest are genuine or due to measurement differences [10].

However, the results need to be interpreted in the context of several limitations. First, as with most cohort studies, there was selective attrition over time, in particular for the ALSPAC cohort which, compared to Pelotas, experienced greater attrition that was also more socially patterned. However, all analyses were performed using FIML estimation which allowed over 9000 participants to be included by excluding participants with no measures but allowing those with missing outcome data to be included under the assumption of missing at random (MAR). We believe that the MAR assumption is plausible given that the variables included in the analysis model were strongly predictive of missing data (particularly the sociodemographic and biological confounders, maternal depression, parental separation and childhood behavioural problems). Second, only measures of lifetime drug use were available in Pelotas meaning that these findings could be due to reverse causality (if illegal drug use began before age 11 years). Third, exposures and outcomes used were assessed using parent-report (behavioural problems) or self-report (all outcomes) meaning that results could be subject to reporting bias. However, all scales used have been validated for use in both the UK and Brazil, and using different reporters for exposure and outcome would have minimised shared-rater bias. Fourth, although the derived latent classes of behavioural problems showed similar profiles across study, there was a bigger within-class probability for the item ‘fights’ in Pelotas compared to ALSPAC, which may have impacted on the strength of the associations with later outcomes. However, there was a theoretical justification for allowing noninvariance for this item (see Online Resource 1), and a high probability of fighting in childhood is most likely to drive the association with later criminal behaviour, which was in fact stronger in ALSPAC compared to Pelotas for conduct problems.

### Comparison with Previous Literature

The higher prevalence of childhood behavioural problems in Brazil compared to the UK in this study supports a systematic review and meta-analysis which found that the average prevalence of behavioural problems in Brazil assessed using the SDQ was 21% [4], compared to 13% in the UK [45]. This could be explained by more Brazilian children living in impoverished urban environments which are associated with multiple risk factors for behavioural problems [4]. Perhaps surprisingly, there was a higher prevalence of criminal behaviour in early adulthood in the UK compared to Brazil. However, this was driven by non-violent crime (with violent crime being more common in Brazil). This supports previous research using the same sample at earlier ages [8] and is consistent with evidence that England lies in a cluster of Anglo-Saxon countries with high levels of property crime and drug use, but lower levels of violence, whereas Brazil may be more similar to a cluster of Eastern European countries, which have high levels of violence but not high levels of non-violent property crime or drug use [5].

Associations between childhood conduct problems and criminal behaviour, emotional disorders and NEET in adulthood have been shown previously in HICs (e.g. [1–3]). Additionally, these findings support a previous study that used both ALSPAC and Pelotas cohorts to show that behavioural problems at age 11 years carry similar risk for crime at age 18 years [8]. As far as we are aware, this is the first study to compare risk for a wider range of outcomes in early adulthood across very different social settings, such as Brazil and the UK. Interestingly, we found that conduct problems were more strongly associated with substance use in early adulthood in Brazil compared to the UK. This could be due to excessive alcohol use and illegal drug use (particularly cannabis) being much more normative in the early twenties in the UK compared to Brazil, meaning that risk factor effects are diluted. This finding supports a previous study using ALSPAC that found weaker associations between different developmental trajectories of childhood conduct problems and alcohol-related problems after age 20 years (Hammerton, unpublished manuscript). In contrast to this, we found that associations between conduct problems and NEET were much stronger in the UK. This could be explained by unemployment being a greater societal problem in Brazil compared to the UK [46] with individual risk factors in childhood having a weaker influence on a young person finding work in early adulthood.

The weak associations found for childhood oppositional problems were surprising given previous research showing strong associations with emotional disorders in both HICs [12–14] and Brazil [15]. One explanation could be the long timespan examined in this study (over 10 years) whereas the previous study in Brazil used a cross-sectional sample of children and adolescents [15]. Additionally, evidence shows that it is ‘irritable’ behaviours such as losing temper anger, and getting easily annoyed that drive the associations with later emotional disorders [12, 15, 47, 48]. In the current study, those with oppositional problems had a high probability of both losing their temper and being disobedient, so we were not able to differentiate between ‘irritable’ and ‘defiant’ behaviours. However, exploratory analyses examining each behaviour separately showed that associations were generally stronger for temper tantrums compared to disobedience, with the exception of drug use and NEET which were stronger for disobedience compared to temper tantrums (results available on request). The importance of specific components of ODD should be investigated further in future studies.

The associations found were not explained by sociodemographic, biological, family and neighbourhood confounders. When additionally adjusting for comorbidity at age 11 years (emotional and hyperactivity problems), all associations weakened as expected, and there was no longer evidence of associations between conduct problems with MDD and GAD. However, these results need to be interpreted with caution given that emotional problems are likely to be on a causal pathway from behavioural problems to these outcomes. Further research is needed to explore the pathways that might explain these associations. These findings could reflect a potentially causal association; for example, conduct problems may lead to potential snares (such as substance use, isolation, peer deviance or curtailed education) that trap young people into experiencing persisting problems. Alternatively, associations (particularly for criminal behaviour and substance use) may reflect the persistence of an underlying theoretical construct, such as behavioural dysregulation and under control [49].

### Conclusions and Future Directions

Children in Brazil and the UK who display both conduct problems and oppositional problems are at high risk for multiple adverse outcomes in early adulthood, and therefore represent an important group to target for interventions. Children with behavioural problems in Brazil are at greater risk of developing problems with substances than those in the UK, whereas those in the UK are at greater risk of becoming unemployed or engaging in criminal behaviour in adulthood. There is a strong evidence-base for the efficacy of preventive interventions early in life that aims to reduce behavioural problems and later criminal behaviour [50]. These prevention strategies often focus on improving social and emotional development in the child and targeting parent factors [50, 51]. However, more research is needed to evaluate the long-lasting impact of these strategies on a wider range of adverse outcomes. Additionally, the evidence-base for the efficacy of these programmes is much scarcer in LMICs [52, 53], and findings from HICs cannot necessarily be generalised to LMICs where socioeconomic factors and health systems differ [54]. Therefore, these early prevention strategies, and their long-lasting impact, also need to be evaluated in Brazil.

Future research should examine whether these findings extend to other LMICs and identify the mechanisms (such as curtailed education or social isolation) that may underlie these strong associations in order to identify potential intervention targets in both middle- and high-income settings.

## Electronic supplementary material

ESM 1 (PDF 249 kb)

ESM 2 (PDF 76 kb)

ESM 3 (PDF 86 kb)

ESM 4 (PDF 79 kb)
