# Supplementary material for: Childhood Behavioural Problems and Adverse Outcomes in Early Adulthood: a Comparison of Brazilian and British Birth Cohorts
Source: J Dev Life Course Criminol. 2019 Oct 27;5(4):517–35. doi: 10.1007/s40865-019-00126-3 (PMC6942009; doi:10.1007/s40865-019-00126-3)
Supplement: Supplementary file 1 — (PDF 249 kb) [file 40865_2019_126_MOESM1_ESM.pdf]

Ttitle: Childhood behavioural problems and adverse outcomes in early adulthood: a comparison of Brazilian and British birth cohorts

Journal: Journal of Developmental and Life-Course Criminology

Authors: Gemma Hammerton (Ph.D.), Joseph Murray (Ph.D.), Barbara Maughan (Ph.D.), Fernando C. Barros (Ph.D.), Helen Gonçalves (Ph.D.), Ana Maria B. Menezes (PhD.), Fernando C. Wehrmeister (Ph.D.), Matthew Hickman (Ph.D.), Jon Heron (Ph.D.)

Dr Gemma Hammerton, Professor Matt Hickman and Dr Jon Heron are with Population Health Sciences, University of Bristol. Professor Joseph Murray, Professor Fernando C. Barros, Professor Helen Gonçalves, Professor Ana Maria B Menezes, and Professor Fernando C. Wehrmeister are with the Postgraduate Program in Epidemiology, Universidade Federal de Pelotas, Pelotas, Brazil. Professor Barbara Maughan is with the MRC Social, Developmental and Genetic Psychiatry Centre, Institute of Psychiatry, Psychology & Neuroscience, King's College London, London, UK. All authors listed meet authorship criteria.

Corresponding author: Gemma Hammerton, Population Health Sciences, University of Bristol, Oakfield House, Bristol, UK, BS8 2BN. Email: gemma.hammerton@bristol.ac.uk; ORCID: 0000-0002-7781-3857

Online Resource 1. Detail on testing for measurement invariance in latent class analysis

To test for measurement invariance of behavioural problems across study, we followed the procedure outlined by Masyn and colleagues (Masyn 2017). First, an unconditional latent class analysis (LCA) was performed for the full sample (Pelotas and ALSPAC cohorts combined) with the five binary items measuring behavioural problems as latent class indicators. LCA assumes that variability in response is due to a latent (unobserved) grouping.

Starting with a single class, a series of models were fitted, and theoretical and statistical steps were taken to decide on the optimal number of latent classes. Fit statistics included (a) the sample-size adjusted Bayesian information criterion (aBIC) (Schwarz 1978), (b) the Bootstrap Likelihood Ratio Test (BLRT) (Nylund et al. 2007) and the Lo, Mendell & Rubin Likelihood Ratio Test (LMR-LRT) (Lo 2001) which assesses the improvement in model fit for each additional class, and (c) bivariate model fit information—a test of the conditional independence assumption—using Pearson's χ^2. Model fit statistics for the one-class to the five-class model are shown in Online Resource 1 Table 1.

Online Resource 1 Table 1. Model fit indices for class enumeration for latent classes of behavioural problems in full sample (Pelotas and ALSPAC cohorts combined); *N* = 11,499

Classes	Parameters	Likelihood	aBIC	Entropy	Smallest class	Bivariate fit	BLRT	LMR-LRT
1	5	-27573	55178	n/a	100%	6370	$p < 0.001$	$p < 0.001$
2	11	-25413	50895	0.63	32%	230	$p < 0.001$	$p < 0.001$
**3**	**17**	**-25249**	**50604**	**0.70**	**17%**	**9**	**$p < 0.001$**	**$p < 0.001$**
4	23	-25223	50589	0.69	8%	1	$p = 0.030$	$p < 0.001$
5	29	-25218	50614	0.71	4%	0	$p = 0.018$	$p = 0.030$

aBIC: sample-size adjusted Bayesian information criterion (lower values indicate preferred models); BLRT: Bootstrap Likelihood Ratio Test; LMR-LRT: Lo, Mendell & Rubin Likelihood Ratio Test (high values indicate no evidence of improvement in fit from model with one less class)

The three-class model was supported by the fit indices and was also theoretically meaningful with one class showing a high probability of all items (conduct problems class; 17%), another class showing a high probability of items related to oppositional defiant disorder (oppositional problems class; 24%), and a final class showing a low probability of all items (Online Resource 1 Figure 1).

Online Resource 1 Figure 1. Latent class profile plots based on an unconditional three-class model in full sample (Pelotas and ALSPAC cohorts combined); N = 11,499

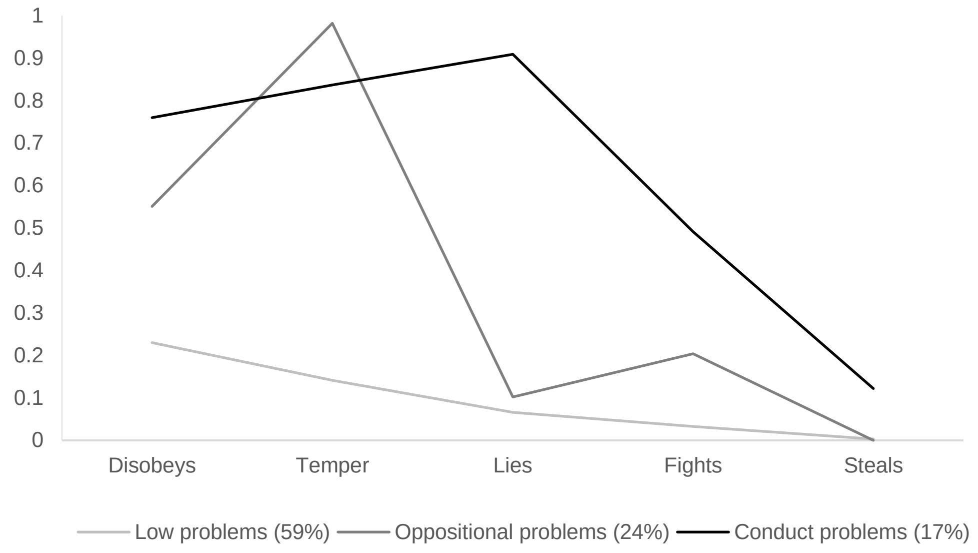

In step 1 an omnibus test of measurement invariance was performed. Two nested models were estimated (see Online Resource 1 Figure 2A and Online Resource 1 Figure 2B). The first model was a three-class latent class multiple indicator multiple cause (MIMIC) model (M1.0) with study (Pelotas or ALSPAC) as a predictor of class membership but no direct effects between study and the latent class indicators. Model M1.0 is the null model with no differential item functioning (DIF). The second model was a three-class latent class MIMIC model (M1.1) with study as a predictor of class membership and class-varying direct effects between study and all the latent class indicators. Model M1.1 is the alternative model with nonuniform DIF for all items.

A number of factors were considered when comparing models. Due to a large sample size (N = 11,499), changes in the sample-size adjusted Bayesian information criterion (aBIC) which takes into account parsimony and sample size as well as fit (with lower values indicating preferred models), effect sizes for direct effects of study on items, and the effect size, w, for the likelihood ratio test statisitic (LRTS) were all considered in combination with the p-value for the LRTS as has been recommended previously (Olivera-Aguilar & Rikoon 2017). As can be seen in Online Resource 1 Table 2 (step 1), model M1.1 (all DIF) provided a much better fit to the data than model M1.0 (no DIF), suggesting that study is a source of DIF for at least one of the five latent class indicators in at least one of the latent classes.

In step 2, individual indicator tests for nonuniform DIF were performed. Bias-adjusted three-step methods (Vermunt 2010) were used (using the modal classes from the original unconditional three-class model and the estimated average classification errors for the modal class assignment) to estimate two models for each of the five latent class indicators (10 models in total). Online Resource 1 Figure 2C and Online Resource 1 Figure 2D show example models for 'disobeys'. Model M2.0.1 (the null, no DIF model) included 'disobeys' as a class indicator and study as a predictor of class membership. Model M2.1.1 additionally included a class-varying direct effect from study to 'disobeys' representing nonuniform DIF for 'disobeys' across study. These models were repeated for each of the four remaining latent class indicators. As can be seen in Online Resource 1 Table 2 (step 2), the nonuniform DIF models provided a better fit to the data for 'disobeys' (model M2.1.1), 'fights' (model M2.1.4), and 'steals' (model M2.1.5) suggesting that these latent class indicators may be functioning differently across study.

In step 3, a latent class MIMIC model including the nonuniform DIF effects for 'disobeys', 'fights' and 'steals' was estimated (model M3.0; Online Resource 1 Figure 2E). ST2 (step 3) shows that model M3.0 provided a better fit to the data than model M1.0 (no

DIF) and did not provide a worse fit to the data (according to the aBIC) than model M1.1 (all DIF).

In step 4, three latent class MIMIC models were estimated. In each of these models, the direct effect from study to one latent class indicator was constrained to be class-invariant (uniform DIF) while the remaining direct effects were class-varying (nonuniform DIF). Online Resource 1 Figure 2F shows a model testing for uniform DIF for ‘disobeys’ (model M4.1). Online Resource 1 Figure 2G shows a model testing for uniform DIF for ‘fights’ (model M4.2). Online Resource 1 Figure 2H shows a model testing for uniform DIF for ‘steals’ (model M4.3). These models were each compared to model M3.0. As can be seen in Online Resource 1 Table 2 (step 4), the models specifying uniform DIF for each item in turn did not provide a worse fit to the data (according to the aBIC) than model M3.0 allowing nonuniform DIF.

In step 5, a latent class MIMIC model including uniform (class invariant) DIF effects for ‘disobeys’, ‘fights’ and ‘steals’ (model M5.0; shown in Figure 1, main text) was compared to model M3.0 (with nonuniform, or class-varying, DIF effects for ‘disobeys’, ‘fights’ and ‘steals’). Online Resource 1 Table 2 (step 5) shows that model M5.0 provided a better fit to the data (according to aBIC) than model M3.0 suggesting that DIF effects for ‘disobeys’, ‘fights’, and ‘steals’ were uniform.

In step 6, the number of latent class indicators with DIF and the magnitude of the DIF effects were considered. The estimated coefficient for the uniform DIF effect of study on ‘disobeys’ was -0.92 (SE = 0.08; $p < 0.001$); OR = 0.40, meaning that within the same latent class, mothers in ALSPAC had 2.5 times the odds of endorsing the ‘disobeys’ item compared to mothers in Pelotas. The estimated coefficient for the uniform DIF effect of study on ‘fights’ was 1.56 (SE = 0.08; $p < 0.001$); OR = 4.75, meaning that within the same latent class, mothers in Pelotas had nearly 5 times the odds of endorsing the ‘fights’ item compared

to mothers in ALSPAC. Finally, the estimated coefficient for the uniform DIF effect of study on ‘steals’ was -1.06 (SE = 0.18; $p < 0.001$); OR = 0.35, meaning that within the same latent class, mothers in ALSPAC had nearly 3 times the odds of endorsing the ‘steals’ item compared to mothers in Pelotas.

The decision that the latent class profiles were comparable across study was based on the number and magnitude of the estimated DIF effects, and the evaluation of the study specific profile plots (shown in Figure 2, main text). It was also considered whether the DIF effects made theoretical sense. The differences seen could be due to cultural differences, for example if punishment is more severe in Brazil compared to the UK, children may be less likely to disobey parents, even when they display behavioural problems. The DIF effects seen for ‘fights’ and ‘steals’ could be a consequence of having a latent class that represents both aggressive and non-aggressive conduct problems, with young people in the UK being more likely to display non-aggressive conduct problems and young people in Brazil being more likely to display aggressive conduct problems (Murray et al. 2015). Additionally, the particulary large DIF effect for ‘fights’ could be due to translation. In ALSPAC, the question asks about “fighting or bullying other children”, whereas in Pelotas the translation of the Brazilian Portuguese is “fighting or frightening other children”.

In step 7, the association between study and class membership was evaluated by comparing model M5.0 (relabeled as model M7.1 in Online Resource 1 Table 2) with a model where all regression coefficients for latent class membership on study were fixed at zero (model M7.0). Online Resource 1 Table 2 (step 7) shows that model M7.0 provides a much worse fit to the data than model M7.1 suggesting that study is related to class membership. Class distribution across study for the partially invariant latent classes (model M7.1) is shown in Figure 2 (main text).

Online Resource 1 Figure 2. Path model representing each analytical step in testing for differential item functioning (DIF) of behavioural problems across study

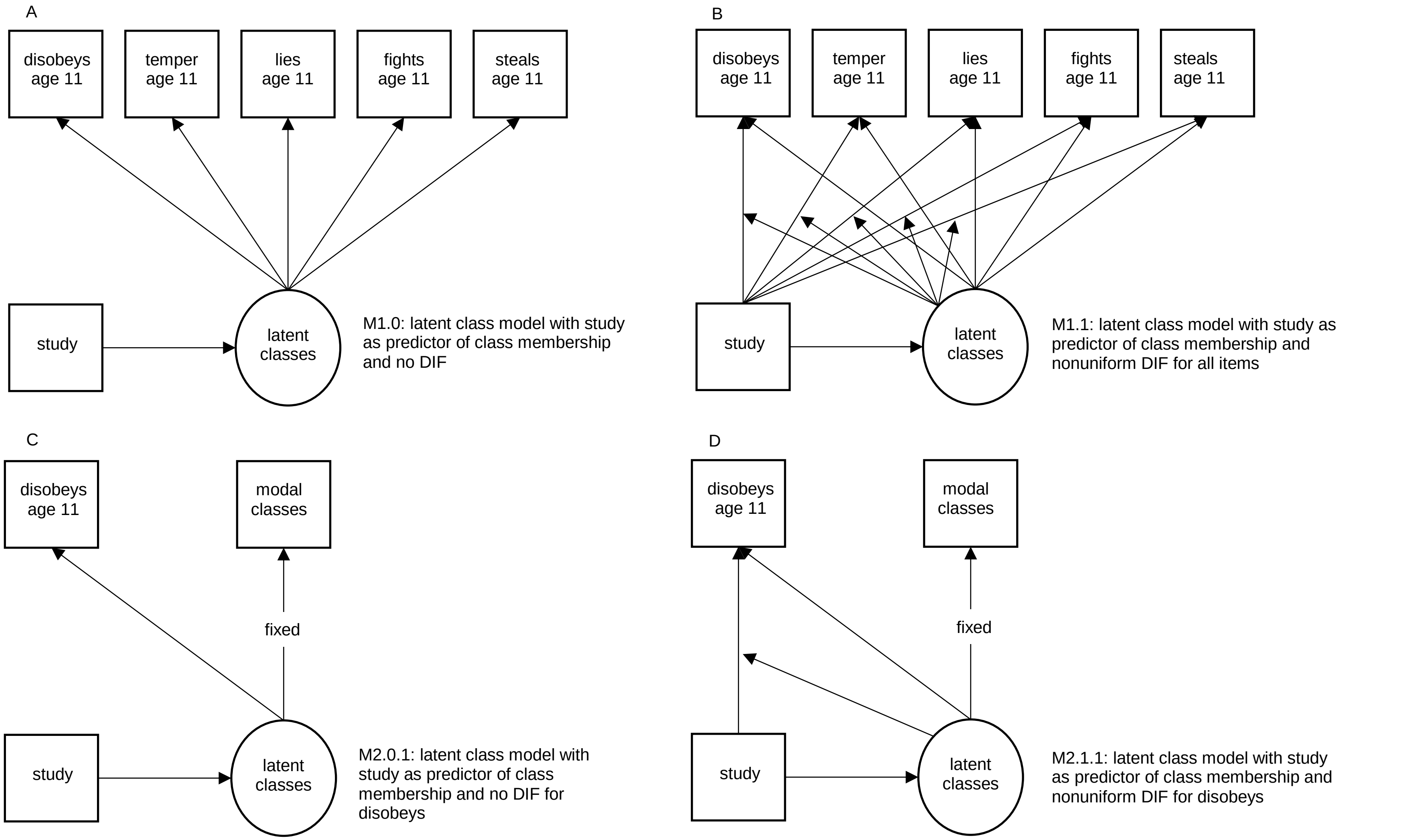

E

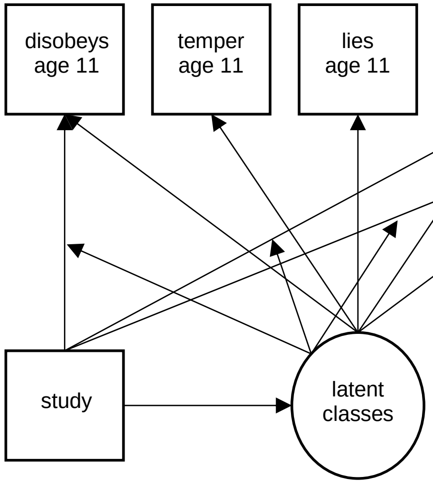

M3.0: latent class model with study as predictor of class membership and nonuniform DIF for disobeys, fights, and steals

F

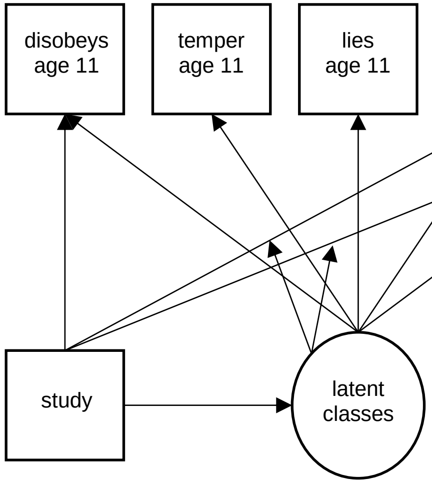

M4.1: latent class model with study as predictor of class membership and uniform DIF for disobeys

G

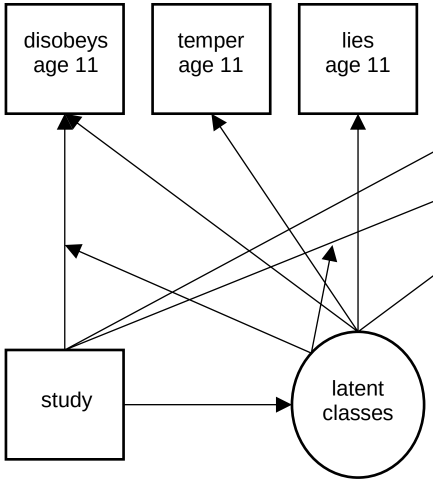

M4.2: latent class model with study as predictor of class membership and uniform DIF for fights

H

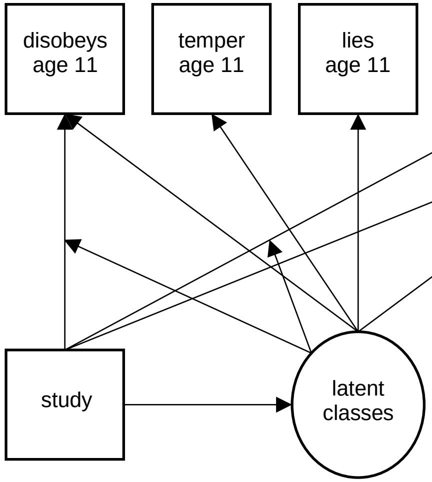

M4.3: latent class model with study as predictor of class membership and uniform DIF for steals

Online Resource 1 Table 2. Model comparisons for differential item functioning (DIF) testing across study with the 3-class multiple indicator multiple cause (MIMIC) model of behavioural problems at age 11 years; *N* = 11,499

Step	Model	Description	aBIC	Likelihood	Parameters	Comparison	LRTS (df)	p	w*
1	M1.0	MIMIC: No DIF	49614	-24748	19	M1.0 vs. M1.1	503.11 (15)	< 0.001	1.21
	M1.1	MIMIC: All DIF	49203	-24497	34				
2	M2.0.1	disobeys: no DIF	36698	-18328	7	M2.0.1 vs. M2.1.1	224.38 (3)	< 0.001	1.21
	M2.1.1	disobeys: nonuniform DIF	36493	-18215	10				
	M2.0.2	temper: no DIF	27174	-13565	7	M2.0.2 vs. M2.1.2	0.27 (3)	0.966	0.001
	M2.1.2	temper: nonuniform DIF	27192	-13565	10				
	M2.0.3	lies: no DIF	26809	-13383	7	M2.0.3 vs. M2.1.3	14.07 (3)	0.003	0.08
	M2.1.3	lies: nonuniform DIF	26814	-13376	10				
	M2.0.4	fights: no DIF	30736	-15346	7	M2.0.4 vs. M2.1.4	562.05 (3)	< 0.001	3.03
	M2.1.4	fights: nonuniform DIF	30192	-15065	10				
	M2.0.5	steals: no DIF	24891	-12424	7	M2.0.5 vs. M2.1.5	44.65 (3)	< 0.001	0.24
	M2.1.5	steals: nonuniform DIF	24865	-12402	10				
3	M3.0	MIMIC: disobeys, fights, steals nonuniform DIF	49200	-24513	28	M1.0 vs. M3.0	469.78 (9)	< 0.001	1.46
						M3.0 vs. M1.1	33.33 (6)	< 0.001	0.13
4	M4.1	MIMIC: disobeys with uniform DIF	49195	-24517	26	M4.1 vs. M3.0	8.18 (2)	0.017	0.05
	M4.2	MIMIC: fights with uniform DIF	49188	-24514	26	M4.2 vs. M3.0	0.48 (2)	0.787	0.003
	M4.3	MIMIC: steals with uniform DIF	49200	-24520	26	M4.3 vs. M3.0	12.51 (2)	0.002	0.08
5	M5.0	MIMIC: disobeys, fights, steals with uniform DIF	49183	-24524	22	M5.0 vs. M3.0	20.27 (6)	0.002	0.08
7	M7.0	MIMIC: C on study@0	49635	-24756	20	M7.0 vs. M7.1	464.16 (2)	< 0.001	3.06
	M7.1	MIMIC: C on study (free)	49183	-24524	22				

*w = $\sqrt{(\frac{LRTS^2}{(N \times df)})}$

Finally, the latent classes with partial measurement invariance (uniform DIF effects for ‘disobeys’, ‘fights’, and ‘steals’) were validated in each study. Validators included heavy alcohol use, fighting, resting heart rate, prosocial behaviour and emotional, hyperactivity, and peer problems at age 11 years. In Pelotas, a self-report questionnaire was used including the questions: ‘did you ever get drunk or feel drunk?’ and ‘in the last year, have you been in a fight where someone got hurt?’ In ALSPAC, children were asked during a focus clinic: ‘during the past two years, have you got really drunk on alcohol?’ and ‘in the past two years, have you been in a fist fight?’ In both studies, pulse rate was recorded (beats per minute; bpm) using a digital monitor (Pelotas: Omron brand, model 711-AC, Beijing, China; ALSPAC: Dinamap 9301 Vital Signs Monitor, UK) while the respondent was seated. To minimise random measurement error, two readings of pulse rate were recorded, and a mean was calculated in each study. Prosocial behaviour, emotional, hyperactivity, and peer problems were assessed using the parent-rated Strengths and Difficulties Questionnaire (SDQ) (Goodman 1997). Associations between the validators and the latent classes (with the ‘low’ class as the reference class) are shown in Online Resource 1 Table 3.

Online Resource 1 Table 3. Validation of the latent classes of behavioural problems across study using related constructs assessed at age 11 years; showing multinomial odds ratio (95% confidence interval) with 'low problems' as the reference class

	Pelotas					ALSPAC					interaction[1]
	n	%	Oppositional problems	Conduct problems	*p value*	n	%	Oppositional problems	Conduct problems	*p value*	*p value*
Alcohol use											
Ever drunk	39	1	3.96 (0.61-25.53)	6.80 (1.32-35.08)	0.057	114	2	1.38 (0.58-3.31)	5.85 (3.15-10.87)	<0.001	0.468
Fighting											
Yes	490	11	1.41 (0.94-2.12)	3.77 (2.70-5.26)	<0.001	771	15	1.85 (1.37-2.49)	3.93 (2.71-5.71)	<0.001	0.572
	mean	SD				mean	SD				
Heart rate	78.89	12.55	0.96 (0.88-1.05)	0.86 (0.78-0.95)	0.008	75.70	11.09	1.04 (0.94-1.14)	1.01 (0.87-1.18)	0.711	0.094
**SDQ scales**											
Prosocial	8.95	1.63	0.59 (0.53-0.67)	0.48 (0.42-0.53)	<0.001	8.37	1.66	0.52 (0.49-0.57)	0.39 (0.35-0.44)	<0.001	0.052
Hyperactivity	4.32	3.10	1.57 (1.48-1.66)	2.16 (2.00-2.33)	<0.001	2.73	2.22	1.80 (1.69-1.91)	2.48 (2.25-2.73)	<0.001	0.006
Emotional	4.20	2.72	1.29 (1.22-1.35)	1.49 (1.41-1.58)	<0.001	1.43	1.71	1.48 (1.38-1.58)	1.73 (1.61-1.87)	<0.001	<0.001
Peer	2.1	1.96	1.39 (1.29-1.50)	1.87 (1.74-2.02)	<0.001	1.05	1.49	1.35 (1.24-1.46)	1.85 (1.71-2.00)	<0.001	0.874

[1] interaction represents whether study (Pelotas vs ALSPAC) modifies the association between validators and behavioural problems

References

Goodman, R. (1997). The Strengths and Difficulties Questionnaire: A Research Note. *Journal of Child Psychology and Psychiatry*, 38(5), 581–586.

Lo, Y. (2001). Testing the number of components in a normal mixture. *Biometrika*, 88(3), 767–778.

Masyn, K.E. (2017). Measurement Invariance and Differential Item Functioning in Latent Class Analysis With Stepwise Multiple Indicator Multiple Cause Modeling. *Structural Equation Modeling*, 24(2), 180–197.

Murray, J., Menezes, A.M.B., Hickman, M., Maughan, B., Gallo, E.A.G., Matijasevich, A. et al. (2015). Childhood behaviour problems predict crime and violence in late adolescence: Brazilian and British birth cohort studies. *Social Psychiatry and Psychiatric Epidemiology*, 50(4), 579–589.

Nylund, K.L., Asparouhov, T. & Muthén, B.O. (2007). Deciding on the number of classes in latent class analysis and growth mixture modeling: A Monte Carlo simulation study. *Structural Equation Modeling*, 14(4), 535–569.

Olivera-Aguilar, M. & Rikoon, S.H. (2017). Assessing Measurement Invariance in Multiple-Group Latent Profile Analysis. *Structural Equation Modeling*, 25(3), 1–14.

Schwarz, G. (1978). Estimating the Dimension of a Model. *The Annals of Statistics*, 6(2), 461–464.

Vermunt, J.K. (2010). Latent class modeling with covariates: Two improved three-step approaches. *Political Analysis*, 18(4), 450–469.
