# Supplementary material for: Childhood Behavioural Problems and Adverse Outcomes in Early Adulthood: a Comparison of Brazilian and British Birth Cohorts
Source: J Dev Life Course Criminol. 2019 Oct 27;5(4):517–35. doi: 10.1007/s40865-019-00126-3 (PMC6942009; doi:10.1007/s40865-019-00126-3)

Title: Childhood behavioural problems and adverse outcomes in early adulthood: a comparison of Brazilian and British birth cohorts

Journal: Journal of Developmental and Life-Course Criminology

Authors: Gemma Hammerton (Ph.D.), Joseph Murray (Ph.D.), Barbara Maughan (Ph.D.), Fernando C. Barros (Ph.D.), Helen Gonçalves (Ph.D.), Ana Maria B. Menezes (PhD.), Fernando C. Wehrmeister (Ph.D.), Matthew Hickman (Ph.D.), Jon Heron (Ph.D.)

Dr Gemma Hammerton, Professor Matt Hickman and Dr Jon Heron are with Population Health Sciences, University of Bristol. Professor Joseph Murray, Professor Fernando C. Barros, Professor Helen Gonçalves, Professor Ana Maria B Menezes, and Professor Fernando C. Wehrmeister are with the Postgraduate Program in Epidemiology, Universidade Federal de Pelotas, Pelotas, Brazil. Professor Barbara Maughan is with the MRC Social, Developmental and Genetic Psychiatry Centre, Institute of Psychiatry, Psychology & Neuroscience, King's College London, London, UK. All authors listed meet authorship criteria.

Corresponding author: Gemma Hammerton, Population Health Sciences, University of Bristol, Oakfield House, Bristol, UK, BS8 2BN. Email: gemma.hammerton@bristol.ac.uk; ORCID: 0000-0002-7781-3857

Online Resource 2. Flow chart of retention in the 1993 Pelotas Birth Cohort Study (left) and the Avon Longitudinal Study of Parents and Children (right)

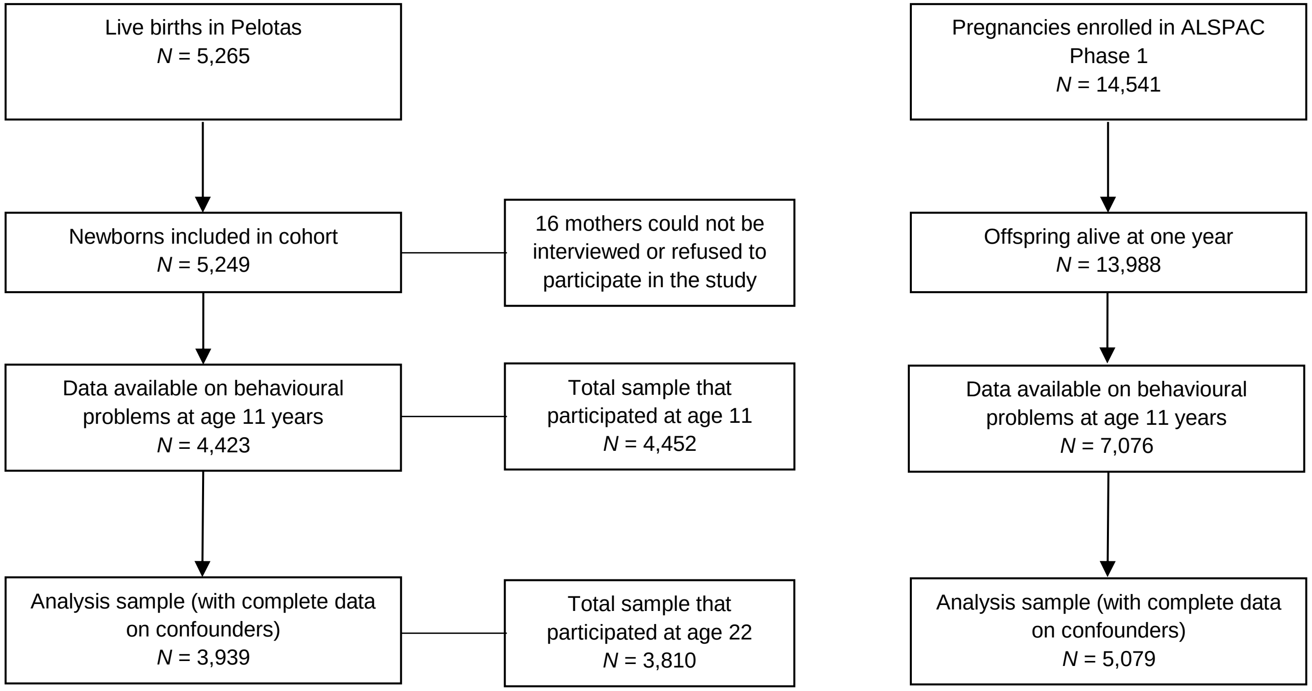

Supplement: Supplementary file 2 — (PDF 76 kb) [file 40865_2019_126_MOESM2_ESM.pdf]
