# Supplementary material for: Childhood Behavioural Problems and Adverse Outcomes in Early Adulthood: a Comparison of Brazilian and British Birth Cohorts
Source: J Dev Life Course Criminol. 2019 Oct 27;5(4):517–35. doi: 10.1007/s40865-019-00126-3 (PMC6942009; doi:10.1007/s40865-019-00126-3)
Supplement: Supplementary file 3 — (PDF 86 kb) [file 40865_2019_126_MOESM3_ESM.pdf]

Title: Childhood behavioural problems and adverse outcomes in early adulthood: a comparison of Brazilian and British birth cohorts

Journal: Journal of Developmental and Life-Course Criminology

Authors: Gemma Hammerton (Ph.D.), Joseph Murray (Ph.D.), Barbara Maughan (Ph.D.), Fernando C. Barros (Ph.D.), Helen Gonçalves (Ph.D.), Ana Maria B. Menezes (PhD.), Fernando C. Wehrmeister (Ph.D.), Matthew Hickman (Ph.D.), Jon Heron (Ph.D.)

Dr Gemma Hammerton, Professor Matt Hickman and Dr Jon Heron are with Population Health Sciences, University of Bristol. Professor Joseph Murray, Professor Fernando C. Barros, Professor Helen Gonçalves, Professor Ana Maria B Menezes, and Professor Fernando C. Wehrmeister are with the Postgraduate Program in Epidemiology, Universidade Federal de Pelotas, Pelotas, Brazil. Professor Barbara Maughan is with the MRC Social, Developmental and Genetic Psychiatry Centre, Institute of Psychiatry, Psychology & Neuroscience, King's College London, London, UK. All authors listed meet authorship criteria.

Corresponding author: Gemma Hammerton, Population Health Sciences, University of Bristol, Oakfield House, Bristol, UK, BS8 2BN. Email: gemma.hammerton@bristol.ac.uk; ORCID: 0000-0002-7781-3857

Online Resource 3. Multivariable associations between potential confounders and the latent classes of behavioural problems at age 11 years; showing multinomial odds ratio (95% confidence interval) with 'low problems' as the reference class

	Pelotas (*N* = 3,939)					ALSPAC (*N* = 5,466)					interaction[1]
**Perinatal**	mean	SD	Oppositional problems	Conduct problems	*p value*	mean	SD	Oppositional problems	Conduct problems	*p value*	*p value*
Biological factors	1.55	1.08	1.19 (1.06-1.33)	1.41 (1.24-1.61)	<0.001	0.72	0.86	1.12 (1.00-1.28)	1.18 (0.99-1.40)	0.023	0.249
Sociodemographic factors	0.8	0.95	1.04 (0.91-1.19)	1.36 (1.18-1.57)	<0.001	0.32	0.65	1.08 (0.92-1.28)	1.43 (1.16-1.76)	0.002	0.882
Sex	n	%				n	%				
Female	1996	51	0.81 (0.64-1.10)	0.62 (0.47-0.81)	0.002	2709	50	0.87 (0.71-1.06)	0.44 (0.30-0.65)	<0.001	0.336
**Family**											
Maternal depression											
Yes	1198	30	3.40 (2.47-4.69)	6.33 (4.59-8.73)	<0.001	645	12	2.19 (1.59-3.00)	2.96 (1.96-4.47)	<0.001	0.008
Parental separation											
Yes	2308	59	1.23 (0.96-1.57)	2.34 (1.70-3.22)	<0.001	1178	22	1.22 (0.93-1.58)	2.24 (1.56-3.21)	<0.001	0.982
**Neighbourhood**											
Fear of neighbourhood											
Yes	473	12	1.32 (0.89-1.97)	1.33 (0.88-2.00)	0.292	332	6	1.03 (0.66-1.61)	1.64 (0.95-2.85)	0.198	0.543

[1] interaction represents whether study (Pelotas vs ALSPAC) modifies the association between potential confounders and behavioural problems
