# Supplementary material for: Childhood Behavioural Problems and Adverse Outcomes in Early Adulthood: a Comparison of Brazilian and British Birth Cohorts
Source: J Dev Life Course Criminol. 2019 Oct 27;5(4):517–35. doi: 10.1007/s40865-019-00126-3 (PMC6942009; doi:10.1007/s40865-019-00126-3)
Supplement: Supplementary file 4 — (PDF 79 kb) [file 40865_2019_126_MOESM4_ESM.pdf]

Title: Childhood behavioural problems and adverse outcomes in early adulthood: a comparison of Brazilian and British birth cohorts

Journal: Journal of Developmental and Life-Course Criminology

Authors: Gemma Hammerton (Ph.D.), Joseph Murray (Ph.D.), Barbara Maughan (Ph.D.), Fernando C. Barros (Ph.D.), Helen Gonçalves (Ph.D.), Ana Maria B. Menezes (PhD.), Fernando C. Wehrmeister (Ph.D.), Matthew Hickman (Ph.D.), Jon Heron (Ph.D.)

Dr Gemma Hammerton, Professor Matt Hickman and Dr Jon Heron are with Population Health Sciences, University of Bristol. Professor Joseph Murray, Professor Fernando C. Barros, Professor Helen Gonçalves, Professor Ana Maria B Menezes, and Professor Fernando C. Wehrmeister are with the Postgraduate Program in Epidemiology, Universidade Federal de Pelotas, Pelotas, Brazil. Professor Barbara Maughan is with the MRC Social, Developmental and Genetic Psychiatry Centre, Institute of Psychiatry, Psychology & Neuroscience, King's College London, London, UK. All authors listed meet authorship criteria.

Corresponding author: Gemma Hammerton, Population Health Sciences, University of Bristol, Oakfield House, Bristol, UK, BS8 2BN. Email: gemma.hammerton@bristol.ac.uk; ORCID: 0000-0002-7781-3857

Online Resource 4. Associations between the latent classes of behavioural problems at age 11 years and adverse outcomes at ages 22 to 24 years after adjusting for perinatal, family and neighbourhood confounders and comorditiy (emotional and hyperactivity problems at age 11 years); showing risk ratio (95% confidence interval) with 'low' problems as the reference group

	Pelotas (*N* = 3,939)				ALSPAC (*N* = 5,079)				interaction[1]
**Model 3**	%	Oppositional problems	Conduct problems	*p value*	%	Oppositional problems	Conduct problems	*p value*	*p value*
Criminal behavioural	10	1.32 (0.88-1.99)	1.64 (1.04-2.57)	0.100	13	0.75 (0.51-1.11)	2.47 (1.76-3.46)	< 0.001	0.003
MDD	5	1.19 (0.64-2.22)	1.32 (0.68-2.56)	0.709	9	1.29 (0.85-1.96)	1.72 (0.96-3.06)	0.133	0.840
GAD	16	0.96 (0.76-1.20)	1.20 (0.92-1.56)	0.177	9	0.94 (0.60-1.47)	1.53 (0.82-2.85)	0.386	0.772
Hazardous alcohol use	21	1.02 (0.85-1.23)	1.21 (0.98-1.51)	0.147	42	1.05 (0.95-1.15)	0.84 (0.63-1.11)	0.226	0.072
Illicit drug use	39	1.11 (1.00-1.23)	1.25 (1.09-1.43)	0.005	59	1.05 (0.99-1.11)	1.07 (0.93-1.22)	0.273	0.267
NEET	22	1.15 (0.97-1.38)	1.23 (0.99-1.52)	0.147	9	1.11 (0.71-1.75)	2.56 (1.62-4.06)	<0.001	0.010

[1] interaction represents whether study (Pelotas vs ALSPAC) modifies the association between behavioural problems and each adverse outcome; MDD=Major Depressive Disorder; GAD=Generalised Anxiety Disorder; NEET=not in education, employment or training
